# Fibroid Tumors of the Uterus

**Published:** 1876-08

**Authors:** J. B. Crandall

**Affiliations:** Sterling, Ill.


### FIBROID TUMORS OF THE UTERUS.

Reported by J. B. CRANDALL, M. D., Sterling, Ill.

Laura King, aged thirty-seven years, never has been pregnant. The patient's early history I give as she gave it to me.

At the early age of eleven years she commenced to have her menstrual periods. She had a moderate flow at each monthly period for some four or five months, when they ceased and did not reappear until about eighteen months later. Her health being rather poor *ad interim*, again at the age of thirteen her health began to improve, her menses reappeared and continued regular for some twelve months. After that her periods averaged from three to five months; her health being very changeable until she was about sixteen. She was then taken down with a fever and had a complication of diseases; was confined to her bed for several months. Her last complication was soreness and great pain over the region of the uterus; said that her attending physician called it inflammation of the womb. But she finally recovered, and at the age of seventeen she got strong again, her periods became quite regular, and she enjoyed tolerably good health, with the exception of more or less trouble from granulated eye lids, up to the date of the development of fibroids in the uterus.

Her attention was first called to a circumscribed spot about two inches below and to the left of the umbilicus, on or about the first of June, 1875, which seemed quite hard to the touch. Mrs. K. was residing at an adjoining town, and called upon a physician who recommended a course of hip baths, blisters, and irritating plasters. She did not improve under this treatment, the tumor increased in size, and her general health was gradually failing.

Her case becoming an alarming one to herself and friends, she was induced to visit Chicago for the purpose of consulting Prof. W. H. Byford, December 13, 1875. After making the necessary examination Dr. Byford gave her a letter with the diagnosis of her case and plan of treatment to hand to her attending physician in Sterling. The contents of the letter are as follows:

CHICAGO, Dec. 15th, 1875.

MY DEAR DOCTOR: Mrs. Arthur King, of Sterling, Illinois, visited me a day or two since. I find her the subject of a fibrous tumor of the uterus that extends almost as high as the umbilicus. It is developed mostly in the right side of the organ. I prescribed for her Squibb's fluid extract of ergot, hoping it might benefit, if it did not cure her. I sent by her a copy of my address before the American Medical Association, in which you will find all that I can say of the treatment of fibrous tumors of the uterus. I cannot advise any better course of treatment than is there given particularly, and I have great faith, as you will see, in the efficacy of ergot in the treatment of these cases.

I am very respectfully,

W. H. Byford.

Mrs. King, upon her return from Chicago, delayed taking the medicine ordered by Prof. Byford for some six or seven days. She took the first dose as ordered, 30 drops of Squibb's fluid extract of ergot on the morning of Dec. 20th, took three doses during the day. Pains commenced soon after taking the second dose. They were excruciatingly severe for some three hours, after which they continued less severely for two days and nights. She had more or less hemorrhage from the uterus after taking the ergot.

Dec. 26th, 1875, made an examination, which confirmed that made by Prof. Byford, only the tumor had risen a little above the umbilicus. The patient was in a state of nervous prostration and worn out by severe pain and loss of sleep. The pulse was very feeble and almost running together, numbering from 110 to 120 per minute; skin dry and hot; temperature 100°, with great pain and tenderness over the uterus and lower bowels. The feet were drawn up; the face bore a pinched and peculiar expression, common in cases of peritoneal inflammation.

Dec. 27th. Ordered morphia sulphate gr. $\frac{1}{4}$, to be repeated every fourth hour. Patient became easier and slept for about four hours after the fourth dose. I also gave fluid extract aconite gtts. iv, in water, to be repeated every third hour.

Dec. 28th. Patient quiet, and the symptoms were all changed for the better. Continued the morphia and aconite for about a week, giving smaller doses and at longer intervals.

Jan. 5th, 1876. All febrile symptoms having subsided the patient was left quite weak. Ordered the following:

℞ Ferri. et Quin. Citrat, . . . . . . . . . . . . . . . . . . . ʒ i.

Acidi Citrici, . . . . . . . . . . . . . . . . . . . . . . . . . ʒ ij.

Spts. Vin. Gallici, } *aa*. . . . . . . . . . . . . . . . . ℥ ij.

Syr. Simpl.,

Dose, teaspoonful four times a day, with nourishing diet, beef tea and bitter ale, to be taken *pro re nata*. To procure rest, ordered a small opii. et camph. pill at bed time.

Jan. 11th. The patient commenced to pass from the uterus small masses of fibrous growths, ranging in size from that of a small chestnut to an English walnut. There were also portions of fleshy substance that came away that evidently belonged to a much larger tumor, as the edges were rough and uneven, showing they had been torn from the main tumor. The odor from those pieces was very offensive. This discharge of foreign growth lasted for about ten days, the smaller ones coming first, which were in the main free from decomposition, but the larger were very offensive and much decayed. The volume of the uturus was much diminished, and as the tenderness and hardness of the organ had abated, the patient was on July 21st ordered to continue the use of the ergot. She had up to this date taken only three doses. It was continued for a week but had no apparent effect except to nauseate and keep her from taking food. Patient had no pains this time from the use of ergot; had a discharge of yellowish matter like thin pus. The ergot was discontinued for one week.

Feb. 1st. Again commenced the use of ergot as first ordered, and continued it for two weeks without much effect, the discharge becoming lighter and less in quantity, and less offensive.

Feb. 14th. Patient is drinking ale; up and about the house; gaining in every respect. The uterus is of normal size and position. She has gained ten pounds in weight; says that she feels quite well, and in fact like a new being.

Feb. 26th. Called and found Mrs. K. about her work, and completely cured.

It appears that the first three doses of ergot taken by this patient were the cause of her recovery. They produced such strong and protracted contractions of the uterus as to break up the larger tumor as well as the adhesions of the smaller ones, which were then set free and discharged, as heretofore described.

